# Applying urinary ultrasound combined with CT to predict the risk of spontaneous ureteral stone passage

**DOI:** 10.1186/s12894-025-01990-6

**Published:** 2025-12-16

**Authors:** MingBin Xu, YunPeng Wei, JiaWen Zhao, ShuMing He, ChengYang Li

**Affiliations:** 1 Department of Urology, The Second Affiliated Hospital of Hainan Medical University, Hainan, China; 2 Department of Urology, The First Affiliated Hospital of Guangxi Medical University, Guangxi, China

**Keywords:** Ureteral stones, Spontaneous stone passage, Urinary ultrasound, Non-contrast computed tomography, Prediction model

## Abstract

**Background:**

We aimed to develop a comprehensive predictive model for spontaneous stone passage (SSP) by integrating parameters from urinary ultrasound, non-contrast computed tomography (NCCT), and clinical markers.

**Methods:**

This retrospective cohort study included 303 patients with unilateral solitary ureteral stones (≤10 mm) who underwent both ultrasound and NCCT before conservative management between July 2023 and July 2025. Demographic, clinical, ultrasound, and NCCT parameters were recorded. Patients were followed for one month to assess SSP (NCCT-confirmed expulsion) versus failure. Univariate and multivariable logistic regression analyses were performed, and model performance was evaluated using receiver operating characteristic (ROC) curves, calibration analysis, and decision curve analysis (DCA).

**Results:**

Of the 303 patients, 191 achieved SSP and 112 failed. Independent predictors of SSP included stone location (middle/lower vs. upper ureter), smaller transverse stone diameter, thinner ureteral wall thickness (UWT), higher ureteral jet frequency (UJF), and greater stone-side ureteral jet velocity (all p < 0.05). The integrated model achieved an AUC of 0.829, outperforming NCCT (0.694) and ultrasound (0.774). Calibration and decision curve analyses (DCA) confirmed good agreement and clinical utility.

**Conclusions:**

Combining ultrasound and NCCT parameters significantly improved prediction of SSP compared with single-modality approaches. This model enables individualized risk stratification, supporting clinical decision-making to reduce unnecessary interventions and optimize outcomes.

## Background

Urolithiasis is a global health burden with rising incidence, requiring substantial healthcare resources for management [1]. Ureteral stones account for ~ 20% of cases and are a frequent cause of emergency admissions due to acute colic and obstructive complications [2]. Conservative treatment with medical expulsive therapy is the first-line strategy for small stones (≤ 10 mm), and 50–75% may pass spontaneously [3, 4]. However, when spontaneous passage fails, delayed intervention can lead to complications. Accurate prediction is therefore essential to balance the risks of unnecessary procedures against the dangers of delayed treatment [5].

Many studies have reported various prognostic factors that predict spontaneous passage of ureteral stones [2, 4, 6–8]. Stone size and location are well-established predictors of passage: smaller and more distal stones tend to pass spontaneously, while larger or proximal stones often require intervention [3]. Imaging plays a central role, with non-contrast computed tomography (NCCT) regarded as the gold standard for stone detection [9], providing detailed anatomic information such as Hounsfield units (Hu) and ureteral wall thickness (UWT) [10]. Ultrasound, while less sensitive for distal stones, offers the advantages of accessibility, lack of radiation, and the ability to assess functional parameters such as ureteral jet frequency (UJF), which reflects ureteral patency and predicts spontaneous stone passage (SSP) [3].

Inflammatory markers including Neutrophil-to-lymphocyte ratio (NLR), CRP, and procalcitonin have been associated with reduced passage rates, reflecting the degree of stone-related ureteral inflammation [7, 11]. Patient factors such as comorbidities, metabolic syndrome, and body mass index (BMI) may further influence outcomes, although their role remains less well defined [4].

Despite these advances, existing predictive models still have constraints. Single-imaging modality may miss useful information. Ultrasound has limited sensitivity in detecting distal ureteral stones; NCCT cannot assess the peristaltic function of the ureters. The current models usually solve the image-based question with respect to a unimodal type of data in isolation rather than by combination, which results in suboptimal accuracy. In addition, few studies have effectively integrated structural and functional measurements to improve prediction [2, 3].

Given that there is currently no relevant research on predicting spontaneous ureteral stone passage through the combination of NCCT and color Doppler ultrasound (ultrasonography), in this context, the purpose of our study was to accurately predict rates for spontaneous ureteral stone passage by combining pertinent data from ultrasonography and NCCT with clinical parameters. In doing so, we hope that this work helps to address the limitations associated with single-modality or single-parameter models for risk stratification by improving predictive accuracy and enhancing personalized management strategies (including avoidance of unnecessary interventions in low-risk patients while facilitating timely intervention among those who are high-clinical-risk). Ultimately, The goal of this approach is to improve patient outcomes while minimizing complications and resource utilization for the management of ureteral stones.

## Methods

### Patient selection

This retrospective cohort study was conducted at the Second Affiliated Hospital of Hainan Medical University. Patients diagnosed with unilateral ureteral stones by both urinary ultrasound and NCCT who underwent conservative treatment between July 2023 and July 2025 were screened for eligibility. The study protocol was approved by the Institutional Ethics Committee and complied with the Declaration of Helsinki.

### Data collection and definition

**Fig. 1:**
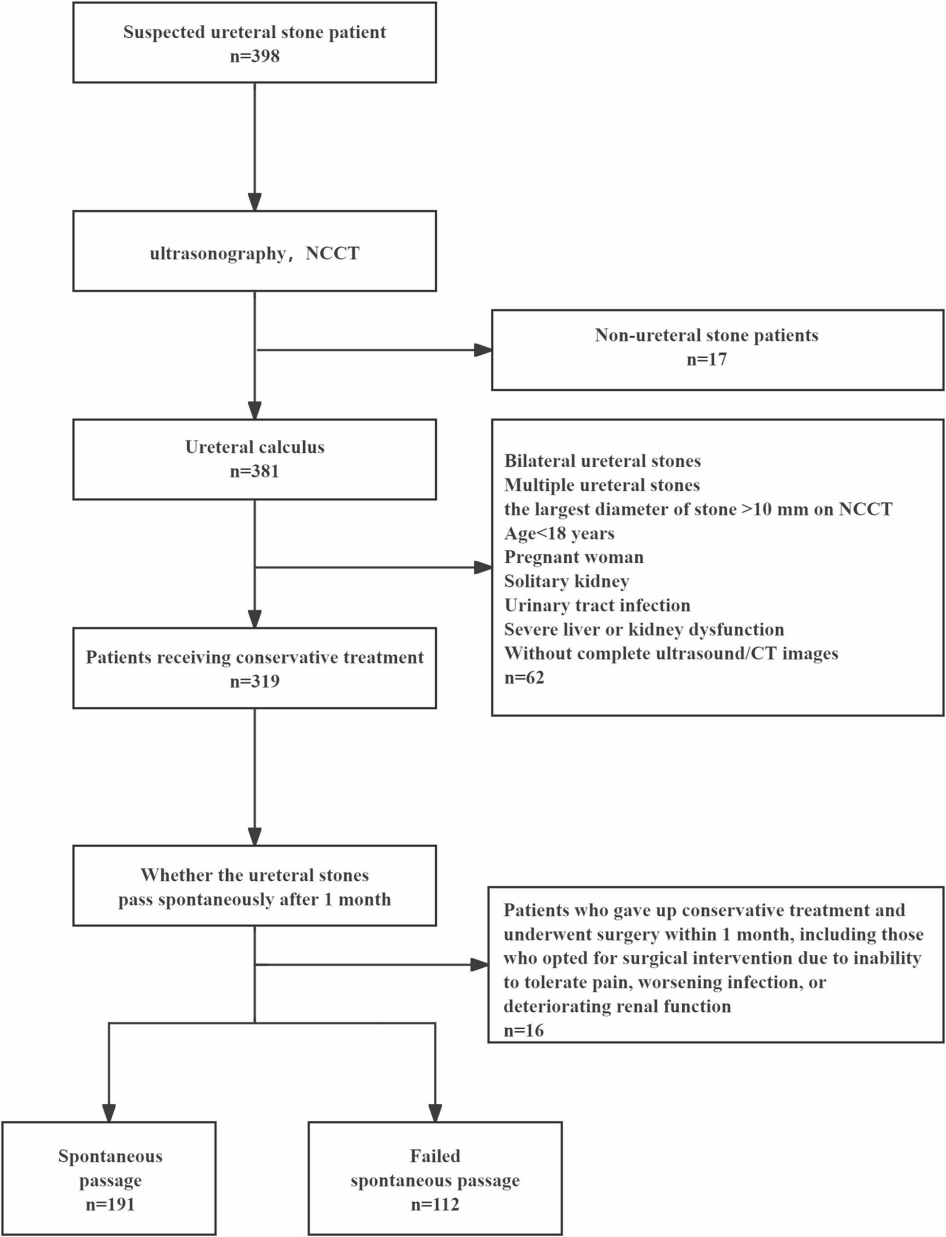
Flowchart of the included study subjects

Inclusion criteria were: (1) patients with unilateral solitary ureteral stones diagnosed by both ultrasound and NCCT; (2) the largest diameter of stone ≦ 10 mm on NCCT; (3) initial conservative treatment with scheduled re-evaluation in 1 month, including using analgesics or α-blockers; (4) complete clinical and imaging data available. Exclusion criteria were: age < 18 years, pregnancy, solitary kidney; severe hepatic or renal dysfunction; incomplete follow-up data and previous urological interventions about the actual stone (Fig. 1).

Demographic and clinical data (age, sex, BMI, comorbidities, stone history, and medication use) were extracted from electronic medical records. Inflammatory markers such as the NLR were measured within 24 h of therapy. Patient functional status was assessed using the Eastern Cooperative Oncology Group Performance Status (ECOG PS) scale [12].

**Fig. 2:**
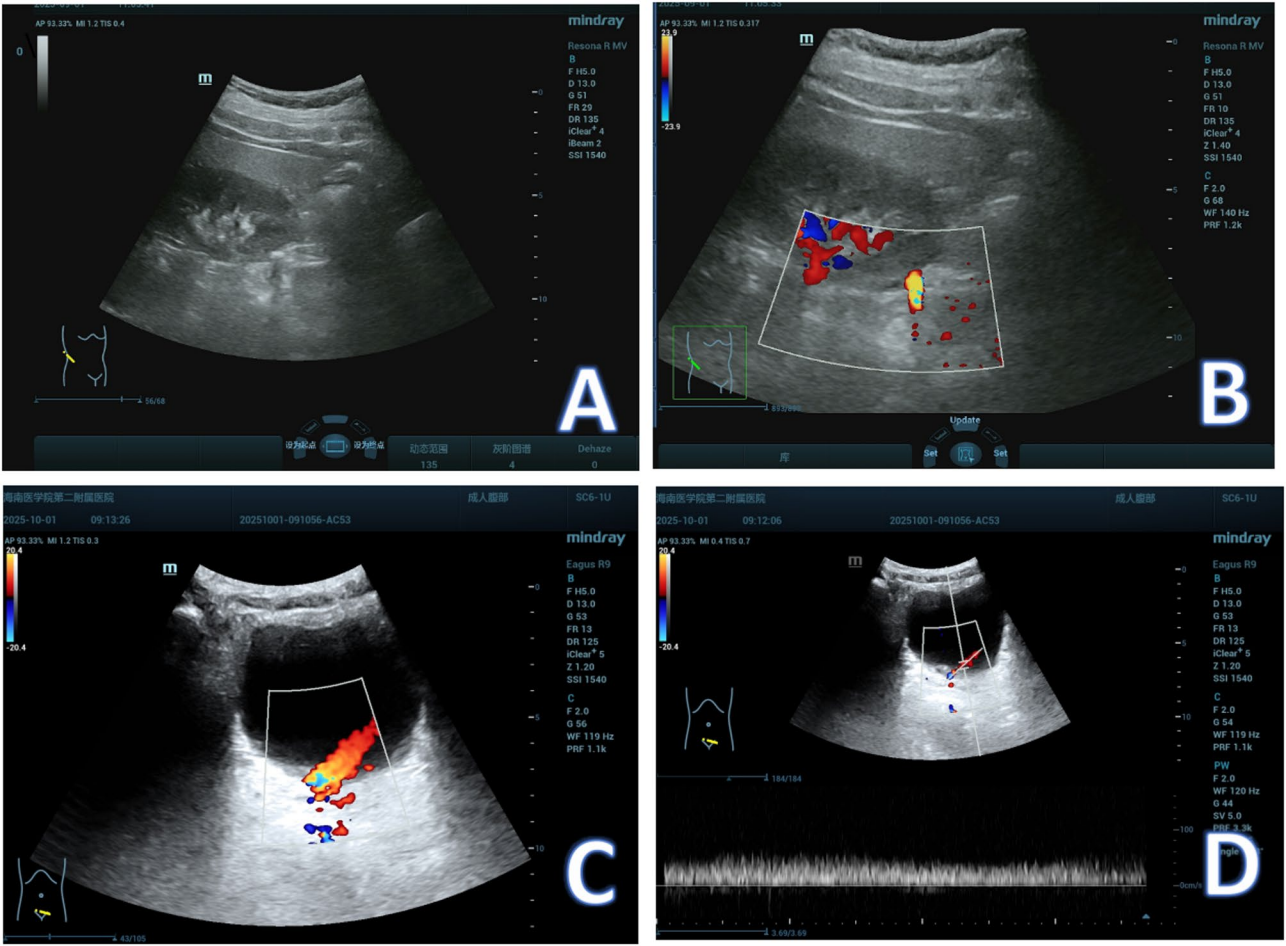
Male, 18 years old, with right upper ureteral stone. (A)longitudinal view of ureteral stone, (B) twinkle artifacts of ureteral stone, (C) Color Doppler image of ureteral squirt, and (D) spectral Doppler image of ureteral jet (jet frequency: 2.0 times/min)

After intake of 500–700 mL of water and a rest period of 30 min, ultrasound examinations were performed (Mindray Eagus R9, C5-1 convex array probe, 1–5 MHz). UJF: At the ureterovesical junction, color Doppler ultrasound was applied to record ureteral jets during a continuous 5-minute observation period. The number of jets was then converted to an average frequency per minute. Jet peak velocity: On the stone-bearing side, color Doppler was applied for 10 min as described by Leung et al. [13]. The peak velocity of three measurable jets was obtained and averaged. To minimize measurement bias, the insonation angle was kept ≤ 60°, with the sampling volume adjusted to cover the ureteral orifice while avoiding bladder wall interference(Fig. 2). All measurements were independently performed by two sonographers with more than five years of urological ultrasound experience. When discrepancies exceeded 15%, a third senior sonographer reviewed the measurement. Interobserver reliability was assessed using a two-way random effects model for the intraclass correlation coefficient (ICC), which showed good agreement for both UJF and jet velocity ≧ 0.80.

NCCT was performed on a Siemens Somatom Definition Flash NCCT scanner (Siemens Healthineers, Erlangen, Germany) using the following parameters: 120 kVp, automatic tube current modulation (auto mAs), slice thickness 1–3 mm, and a dedicated high-resolution stone reconstruction kernel. UWT was measured at the stone site as the maximum distance from the luminal surface to the outer ureteral wall, on an axis perpendicular to the ureteral lumen. Two independent radiologists, each with more than five years of experience in genitourinary imaging, performed all measurements blinded to clinical data. The average of their measurements was used for analysis, and interobserver reliability was assessed using the ICC.

### Study endpoint

The patients were then monitored for 1 month. The SSP was defined as no stone detected on follow-up NCCT. Failure of SSP: persistent imaging stone visualization by NCCT.

### Statistical analysis

Data were analyzed using R software (version 4.3.1) and SPSS version 26.0 (IBM, Armonk, NY). Continuous variables were expressed as mean ± SD or median [IQR] depending on distribution and compared using Student’s t-test or Mann–Whitney U test. Categorical variables were expressed as counts and percentages and compared using χ² or Fisher’s exact test. Univariate logistic regression was first applied to identify factors associated with SSP. Significant variables were entered into multivariable logistic regression to identify independent predictors. Based on these, a nomogram was constructed. Internal validation was performed using 1,000 bootstrap resamplings. Model performance was assessed by: Discrimination: ROC curve and AUC; Calibration: calibration plots; Clinical utility: decision curve analysis (DCA). Statistical significance was defined as *p* < 0.05. 

## Results

A total of 303 patients with unilateral ureteral stones were included (Table 1), of whom 191 (63.0%) achieved SSP and 112 (37.0%) failed. Baseline demographic and clinical variables such as age, sex, BMI, comorbidities, laterality, and stone history did not differ significantly between the two groups (all *p* > 0.05). Compared with the failed group, the SSP group had a significantly higher proportion of lower ureteral stones (67.0% vs. 44.6%, *p* < 0.001), less severe hydronephrosis (40.8% vs. 56.3%, *p* = 0.009), smaller transverse stone diameter (4.6 ± 1.9 mm vs. 5.2 ± 2.0 mm, *p* = 0.013), thinner maximal UWT (2.0 ± 1.4 mm vs. 2.4 ± 1.2 mm, *p* = 0.007), higher UJF (2.9 ± 1.1/min vs. 1.9 ± 1.1/min, *p* < 0.001), and greater jet flow velocity (27 ± 10 cm/s vs. 22 ± 10 cm/s, *p* < 0.001).

**Table 1:** Patients' characteristics according to spontaneous ureteral stone passage status

	SSP Group(n=191)	Failed Group(n=112)	p-value
Age (years)	48±15	47±13	0.467
Gender, n%			0.878
Male	104(54.5%)	62(55.4%)	
Female	87(45.5%)	50(44.6%)	
BMI (kg/m^2^)	23±5	23±5	0.659
Stone location, n%			﹤0.001
Upper	20(10.5%)	38(33.9%)	
Middle	43(22.5%)	24(21.4%)	
Lower	128(67.0%)	50(44.6%)	
Laterality, n%			0.976
Right	73(38.2%)	43(38.4%)	
Left	118(61.8%)	69(61.6%)	
Hydronephrosis, n%			0.009
Grade 0/1/2	113(59.2%)	49(43.8%)	
Grade 3/4	78(40.8%)	63(56.3%)	
Longitudinal diameter of stone (mm)	7.0±2.3	7.4±2.3	0.174
Transverse diameter of stone (mm)	4.6±1.9	5.2±2.0	0.013
Stone density (HU)	633±231	617±239	0.575
Maximal UWT (mm)	2.0±1.4	2.4±1.2	0.007
Presence of stone history, n%	73(38.2%)	39(34.8%)	0.554
The history of SSP, n%	39(20.4%)	25(22.3%)	0.695
Diabetes mellitus, n%	34(17.8%)	23(20.5%)	0.557
Hypertension, n%	51(26.7%)	29(25.9%)	0.877
Coronary artery disease, n%	32(16.8%)	17(15.2%)	0.719
NLR	2.32±0.58	2.28±0.61	0.632
kidney parenchymal thickness (mm)	26±7	25±5	0.501
Alpha-blocker usage, n%	146(76.4%)	90(80.4%)	
Shape, n%			0.232
Oval	113(59.2%)	74(66.1%)	
Irregular	78(40.8%)	38(33.9%)	
UJF	2.9±1.1	1.9±1.1	﹤0.001
Jet flow (side with the stone) (cm/s)	27±10	22±10	﹤0.001
ECOG PS score, n%			0.105
0	157(82.2%)	87(77.7%)	
1	15(7.9%)	19(17.0%)	
2	11(5.8%)	4(3.6%)	
3	6(3.1%)	1(0.9%)	
4	2(1.0%)	1(0.9%)	
duration of medical history (months)	3.5±2.2	3.5±2.0	0.865

UWT, ureteral wall thickness; NLR, Neutrophil-to-lymphocyte ratio; UJF, Ureteral jet frequency; SSP, spontaneous stone passage

Univariate logistic regression identified stone location, hydronephrosis, transverse stone diameter, UWT, UJF, and ureteral jet velocity as significant predictors of SSP (Table 2). In multivariable analysis, five independent predictors remained: stone location (lower vs. upper ureter: OR = 4.706, 95% CI 2.272–9.750, *p* < 0.001; middle vs. upper: OR = 2.844, 95% CI 1.232–6.566, *p* = 0.014), transverse diameter (OR = 0.761, 95% CI 0.653–0.887, *p* < 0.001), UWT (OR = 0.779, 95% CI 0.626–0.969, *p* = 0.025), UJF (OR = 2.702, 95% CI 1.961–3.725, *p* < 0.001), and ureteral jet velocity (OR = 1.064, 95% CI 1.033–1.096, *p* < 0.001). Hydronephrosis, although significant in univariate analysis, was not retained in the multivariable model.

**Table 2:** Univariate and multivariate analysis of the factors associated with SSP

Variables	Univariate regression		Multivariate regression	
	OR(95%CI)	P value	OR(95%CI)	P value
Stone location		﹤0.001		﹤0.001
Upper	Reference	-	Reference	-
Middle	3.404(1.630-7.111)	0.001	2.844(1.232-6.566)	0.014
Lower	4.864(2.584-9.155)	﹤0.001	4.706(2.272-9.750)	﹤0.001
Hydronephrosis		0.010		0.706
Grade 0/1/2	Reference	-	Reference	-
Grade 3/4	0.537(0.335-0.861)		0.895(0.502-1.595)	
Transverse diameter of stone (mm)	0.860(0.762-0.970)	0.014	0.761(0.653-0.887)	﹤0.001
Maximal UWT (mm)	0.786(0.658-0.939)	0.008	0.779(0.626-0.969)	0.025
UJF	2.358(1.797-3.092)	﹤0.001	2.702(1.961-3.725)	﹤0.001
Jet flow (cm/s)	1.051(1.025-1.077)	﹤0.001	1.064(1.033-1.096)	﹤0.001

**Fig. 3:**
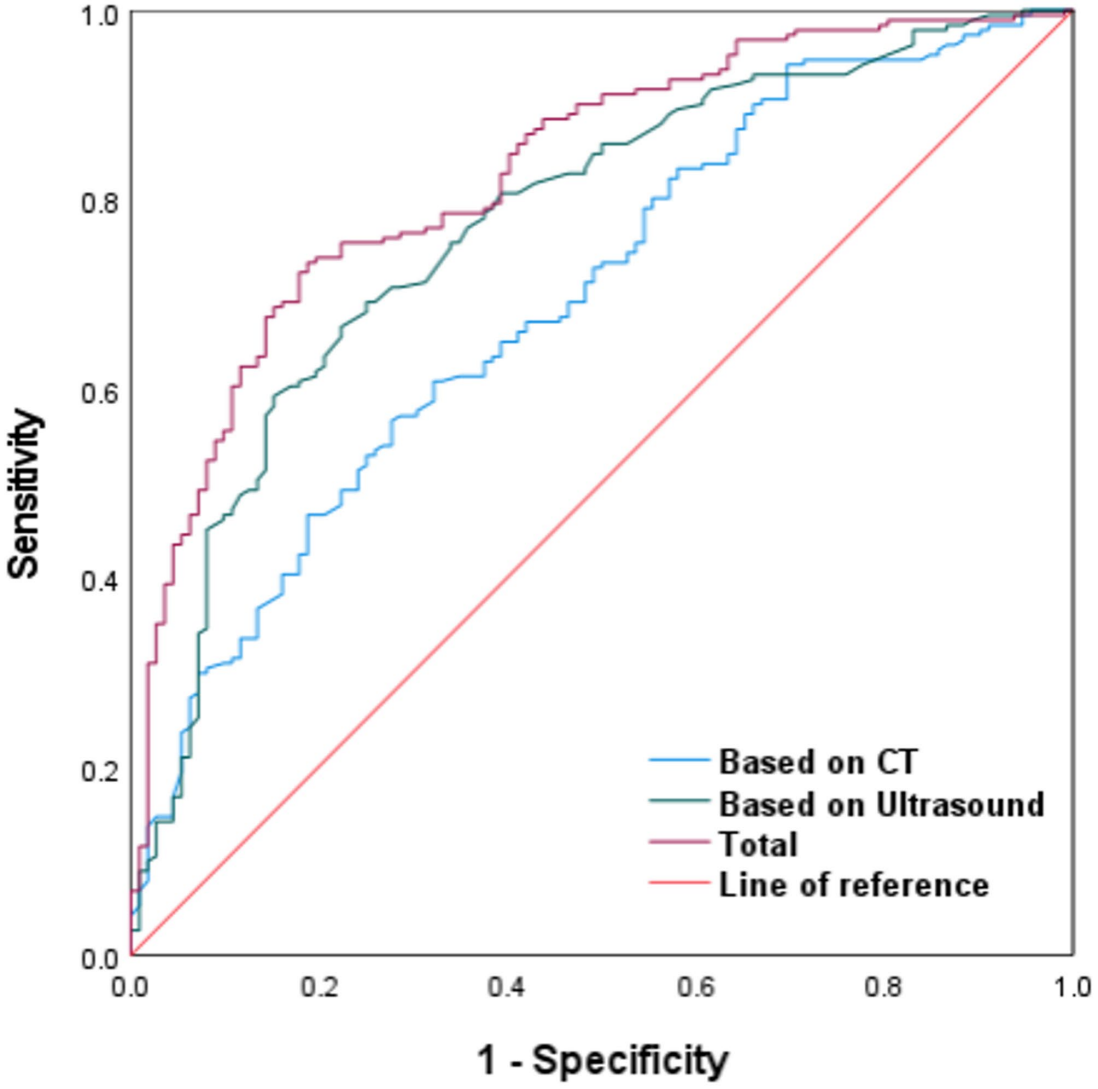
The ROC curve for predicting SSP achieved an AUC of 0.829, with sensitivity of 73.3%, and specificity of 81.2%. For comparison, the AUC values of CT and Ultrasound were 0.694 and 0.774, respectively

**Fig. 4:**
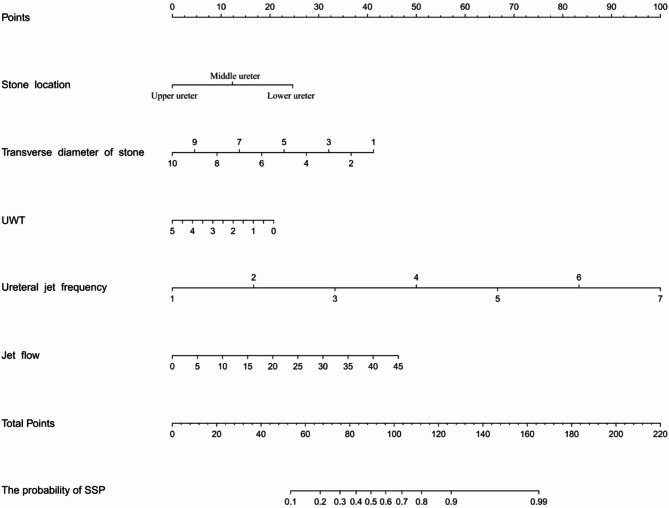
A nomogram based on variables to predict the risk of SSP. To illustrate the practical use of the nomogram (Fig. 4) for predicting SSP, we present a typical clinical case and stepbystep application of the tool: A 45-year-old male patient was admitted to the urology department with rightsided flank pain. Imaging and clinical evaluations yielded the following key data: Stone location: Lower ureter (confirmed by NCCT); Transverse diameter of stone: 4.0 mm (measured via NCCT); UWT: 2.0 mm (assessed by NCCT); Ureteral jet frequency: 3.0 times per minute (detected via color Doppler ultrasound); Ureteral jet flow (stone side): 28 cm/s (measured by Doppler ultrasound). Step 1: Map each variable to its corresponding “Points” on the nomogram. Using the horizontal axes of Fig. 4 (labeled with each predictor), locate the patient’s value for each variable and draw a vertical line upward to the “Points” axis to obtain the variable specific score: Stone location: “Lower ureter” corresponds to 25 points; Transverse diameter of stone: 4.0 mm corresponds to 28 points; Maximal UWT: 2.0 mm corresponds to 12.5 points; Ureteral jet frequency: 3.0 times/min corresponds to 33 points; Jet flow: 28 cm/s corresponds to 28 points; Step 2: Calculate the “Total Points” Sum the points from all variables: Total Points = 25 (stone location) + 28 (transverse diameter) + 12.5 (UWT) + 33 (ureteral jet frequency) + 28 (ureteral jet flow) = 126.5 points; Step 3: Predict the probability of SSP. On Fig. 4, locate “126.5 points” on the “Total Points” axis, then draw a vertical line downward to the “Probability of SSP” axis. This patient’s total points correspond to an ~ 90% probability of SSP. Clinical Interpretation Based on the nomogram’s prediction, this patient has a high likelihood of SSP. Clinicians could therefore prioritize conservative management rather than immediate invasive intervention, aligning with the predictive value of the model

**Fig. 5:**
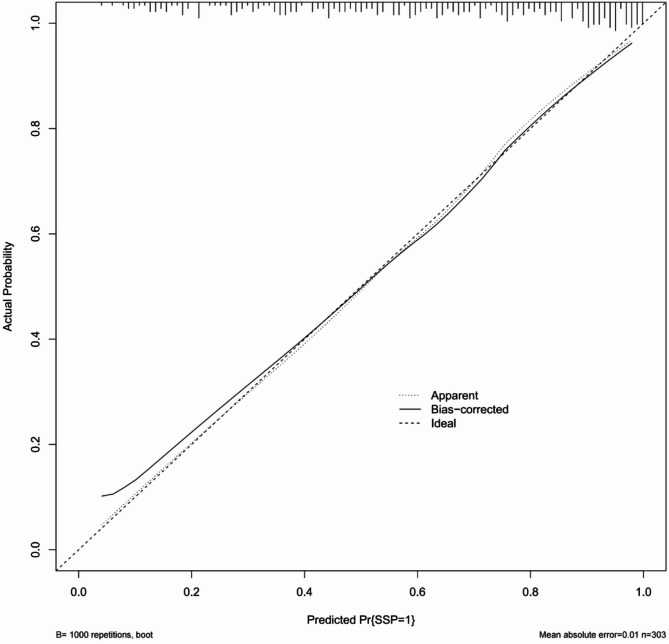
Internal validation calibration plot

**Fig. 6:**
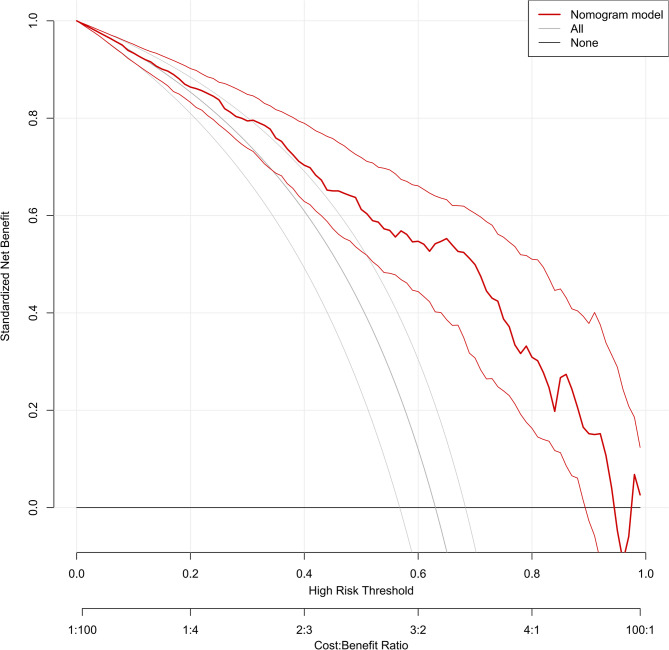
Decision curve analysis: a model for predicting SSP

The integrated model achieved an AUC of 0.829, higher than that of CT (0.694) or ultrasound alone (0.774) (Fig. 3). The calibration plot (Fig. 5 ) demonstrated excellent agreement between predicted and observed outcomes (mean absolute error = 0.01). Decision curve analysis (Fig. 6 ) confirmed a net clinical benefit across a wide range of threshold probabilities (0.1–0.9). A nomogram based on the five independent predictors was constructed to facilitate individualized clinical application (Fig. 4 ).

## Discussion

This study proposes an integrated assessment system that combines urinary ultrasound and NCCT to predict spontaneous ureteral stone passage. Our results demonstrate that the combined approach achieves superior diagnostic performance compared with either modality alone, highlighting the complementary value of structural and functional imaging parameters. Collectively, previous findings highlight the complementary roles of ultrasound and NCCT. While each modality provides valuable predictive insights independently, their integration overcomes the inherent limitations of single-modality evaluations. The combined use of urinary ultrasound and NCCT to predict spontaneous ureteral stone passage is a novel approach, which has implications on current clinical decision-making by supplementing important data lacking from the existing literature.

Ultrasound-derived parameters, as highlighted by Wang et al. [3], provide unique insights into ureteral physiology by assessing real-time functional dynamics rather than static anatomy. Previous study identified stone length, distal location, and UJF as independent predictors of spontaneous passage, with a model AUC of 0.814 [3]. Similarly, Ongun et al. [14] confirmed that a ureteral jet flow peak velocity >15.25 cm/s predicts spontaneous passage with 85.4% sensitivity and 63.1% specificity, underscoring ultrasound’s role in assessing dynamic ureteral function. These findings indicate that ultrasound excels in capturing functional impairment, such as reduced UJF or jet velocity, which reflect compromised ureteral motility due to stone obstruction.

In contrast, NCCT offers precise anatomical characterization, including stone size, density, and ureteral wall thickness, which are closely linked to impaction and passage potential. Mohammadinejad et al. [15] showed that both manual and automated measurements of stone size predict passage with AUCs of 0.82–0.83, highlighting NCCT’s precision in quantifying stone dimensions. Khan et al. [8] further identified UWT as a robust independent predictor, with a cutoff of 1.95 mm yielding 95% sensitivity and 83% specificity (AUC = 0.94), as UWT correlates with inflammation and stone impaction [16]. Additionally, Selvi et al. [4] noted that CT-derived ratios (ureter to stone diameter) and UWT enhance predictive accuracy by reflecting structural obstruction.

When integrated, ultrasound and NCCT complement each other: NCCT cannot assess real-time ureteral function, which is captured by ultrasound, while ultrasound may miss small or distal stones obscured by bowel gas that are clearly visualized on CT. Thus, a distal stone, thinner UWT (measured by NCCT) that had higher UJF and Jet flow could have substantially higher odds of passage than an equally specified alternative positioned proximally but weaker in terms of the same feature: this detail might not be detected by one-image models.

Each of the five predictors identified in our model has shown discriminative value in prior studies; however, their integration into a single multimodal framework yielded improved performance beyond any isolated factor. For instance, Wang et al. [3]. demonstrated that a model incorporating distal location, stone size, and UJF achieved an AUC of 0.814, while Ongun et al. [14]. reported an AUC of 0.747 based on ureteral jet flow velocity. CT-derived parameters have also shown strong predictive power, with Mohammadinejad et al. [15]. reporting AUC values of 0.82–0.83 for stone size, and Khan [8] et al. identifying UWT as a robust marker with an AUC of 0.94. Similarly, Gao et al. [6] proposed a CT-based nomogram with a C-statistic of 0.77. When compared with these prior results, our integrated model achieved an AUC of 0.829, which not only matches but slightly exceeds the upper range of performance from single-modality or single-factor approaches. Importantly, our study demonstrates that combining structural information from NCCT (stone location, diameter, UWT) with functional insights from ultrasound (UJF, jet flow velocity) produces a synergistic effect, enhancing predictive accuracy beyond what has been reported for isolated predictors. Thus, while individual factors may achieve high discriminative value in specific contexts, their integration in a multimodal framework offers a more robust and clinically applicable tool for risk stratification in SSP.

Previous studies provided the strongest evidence for the impact of stone location on SSP [4, 6]. Stone location remains one of the strongest determinants of SSP, with multiple studies consistently showing that distal ureteral stones have substantially higher spontaneous passage rates than proximal or mid-ureteral stones. Coll et al. reported that stones located in the distal ureter had significantly higher passage rates than those in the proximal ureter, independent of stone size [17]. Similar findings were observed in prospective and retrospective cohorts, showing that the closer the stone lies to the ureterovesical junction, the greater the likelihood of expulsion [18, 19]. This phenomenon can be explained by anatomical and physiological factors: the distal ureter is influenced by bladder dynamics, and micturition-related pressure and flow changes may facilitate the migration of stones into the bladder, thus completing passage [20]. Therefore, stone location should be carefully considered when selecting between conservative and active intervention strategies.

Thickened UWT reflects inflammatory or fibrotic changes at the impaction site, narrowing the ureteral lumen and impairing peristalsis, thereby reducing the likelihood of stone passage [21]. UWT as an independent predictor, where thicker walls reflect edema, inflammation, or scarring from prolonged stone irritation. This thickening narrows the ureteral lumen, increases friction between the stone and wall, and impairs peristalsis, mechanisms echoed in Dean et al. [16], which links UWT to reduced motility and increased SSP failure.

Reduced UJF signifies impaired ureteral peristaltic activity, limiting the contractile force required for stone expulsion and increasing the risk of persistent obstruction. A low frequency means reduced contractility of the ureteral smooth muscle, so chronic ischemia or inflammation due to obstruction. The impact would also be softened, reducing the necessary kinetic energy to drive a stone into the bladder and thus directly increasing risk of SPP failure. Reduced jet flow velocity means obstructive disturbance of the urine dynamics. As shown in Ongun et al. [14], More simply, when the velocity of flow is low it means that less urine goes between past the stone so as to reduce hydraulic force for pushing off calculus. This is compounded by obstructive buildup of upstream pressure, which further attenuates streamline velocity and diminishes the “push” required for stone expulsion thus associating low velocities with retention time [20].

In our study, ultrasound-derived parameters demonstrated a higher AUC than CT parameters. One possible explanation is that the mean stone size in our cohort was relatively small (4.8 ± 2.0 mm). For stones < 5 mm, functional assessment of ureteral dynamics by color Doppler ultrasound may provide more precise prediction of SSP than static anatomical measurements obtained by CT. In addition, ultrasound can capture real-time physiological changes such as ureteral peristalsis and urinary flow, which are not reflected in CT imaging. Furthermore, the relatively narrow range of stone size in our cohort may have limited the discriminative power of CT-based parameters (such as diameter or density), thereby favoring ultrasound-based predictors.

A major innovative aspect of our study lies in the integration of structural parameters from NCCT (stone location, stone transverse diameter, and ureteral wall thickness) with functional parameters from urinary ultrasound (UJF and jet flow velocity) into a single predictive model. While previous models have relied predominantly on unimodal imaging features, our approach provides a more comprehensive assessment of both anatomical obstruction and dynamic ureteral function. This dual-modality framework not only improves predictive accuracy but also offers mechanistic insights by linking impaired ureteral physiology with stone impaction. From a clinical standpoint, the nomogram generated in this study provides a practical, individualized tool that can be readily applied at the bedside. It allows urologists to identify patients who are highly likely to achieve spontaneous stone passage and can safely continue conservative management, while at the same time flagging high-risk patients who may benefit from early intervention. Such stratification has the potential to reduce unnecessary surgical procedures, avoid radiation exposure from repeated imaging, minimize complications of delayed treatment, and ultimately optimize resource allocation in urolithiasis care.

Several limitations of this study should be acknowledged. First, it was conducted at a single center with a retrospective design, which may introduce selection bias and limit the generalizability of the findings. Second, the follow-up period was limited to one month. Although short-term outcomes are clinically relevant, delayed stone passage beyond this timeframe may have been missed, potentially underestimating the true spontaneous passage rate. Third, the study cohort predominantly consisted of patients with relatively small stones (mean transverse diameter 4.8 mm), which may have amplified the predictive value of ultrasound-derived functional parameters while attenuating the impact of CT-based measurements. Thus, the performance of the model in patients with larger or more complex stones remains uncertain. Fourth, ultrasound-derived variables such as UJF and flow velocity are operator-dependent and subject to interobserver variability, which may affect reproducibility in different clinical settings despite the good ICC observed in our study. Fifth, although several clinical and inflammatory markers (e.g., NLR, comorbidities) were collected, they were not integrated into the final model due to limited statistical contribution in this dataset. Future studies should further explore multimodal integration of biochemical and anatomical-functional predictors. Sixth, the model did not incorporate detailed ureteral anatomical characteristics (such as curvature, caliber variation, or intramural length) or more comprehensive functional parameters (such as peristaltic coordination and intraluminal pressure), which are known to influence stone impaction and expulsion. Finally, only internal validation using bootstrap resampling was performed. The absence of external validation limits the strength of the conclusions, and independent datasets are required to confirm the robustness and clinical utility of the proposed nomogram.

## Conclusions

To our knowledge, this is among the first attempts to develop a multimodal imaging-based model for predicting ureteral stone passage. By leveraging the complementary strengths of CT and ultrasound, the model achieved higher predictive performance than either modality alone. Although external validation and further exploration with artificial intelligence are warranted, our findings demonstrate that combined ultrasound/NCCT risk stratification offers significant clinical promise, either as a standalone tool or as a complement to existing predictive models.

## Data Availability

The clinical data generated during the current study are available from the corresponding author upon reasonable request.

## Abbreviations

SSP: spontaneous stone passage
NCCT: non-contrast computed tomography
ROC: receiver operating characteristic
UWT: ureteral wall thickness
UJF: ureteral jet frequency
NLR: Neutrophil-to-lymphocyte ratio
BMI: body mass index
AUC: area under the curve
HU: Hounsfield unit
